# Supplementary material for: Tongue thickness measured by ultrasonography is associated with tongue pressure in the Japanese elderly
Source: PLoS One. 2020 Aug 7;15(8):e0230224. doi: 10.1371/journal.pone.0230224 (PMC7413413; doi:10.1371/journal.pone.0230224)
Supplement: Attachment — Submitted filename: Comments of reviewer.docx [file pone.0230224.s001.docx]

In this study, the authors evaluate the thickness of the tongue as an expression of “oral frailty” and as possible swallowing disorder.

**Abstract.**

Page 3, lines 33, 34. The sentence: “The tongue ultrasonography provides a less invasive technique for determining tongue thickness and related aspiration risks for elderly patients”. The authors should better explain or change the second part of this claim. Does the tongue ultrasonography provide a technique for determining aspiration risks?

**Introduction.**

Page 4, lines 56, 57. VF is not performed in some centers, but currently it is performed routinely in many hospitals. You should change the sentence: “…However, VF is a specialized method that is not routinely performed with the general population...”.

Page 4, lines 57, 58. “Hence, several simple and non-invasive techniques have been developed for evaluating swallowing function…”. A non invasive techniques for evaluating swallowing function is the Fiberoptic Endoscopic Evaluation of Swallowing (FEES) that is a gold standard for evaluation of pharyngeal phase of swallowing. You should add this exam with its bibliography to the introduction.

Page 4, lines 59, 60. You should describe some instrumental methods of measuring the tongue pressure with the relative bibliography.

**Mathods. Subjects.**

Page 5, lines 82-85. What are the inclusion and exclusion criteria? What does it mean: “chronic disease”? “We excluded patients who had a history of otorhinolaryngologic disease”. Which ENT diseases have been excluded?

**Statistical analysis.**

In your study how the sample size estimation and power analysis was calculated?

**Discussion.**

Page 15, lines 250, 251. “One serious concern related to oral frailty is that elderly people often developed aspiration pneumonia”. You should better explain how oral frailty can develop aspiration pneumonia. What are the pathophysiological mechanisms? Can oral frailty cause aspiration pneumonia even in elderly people without neurodegenerative diseases? Is this pathological mechanism present in presbyphagia? The alteration of the oral phase of swallowing and the consequent premature spillage, can be the cause of increased risk of aspiration in the elderly people? You should better explain these mechanisms and add the related references.
