# Supplementary material for: Tongue thickness measured by ultrasonography is associated with tongue pressure in the Japanese elderly
Source: PLoS One. 2020 Aug 7;15(8):e0230224. doi: 10.1371/journal.pone.0230224 (PMC7413413; doi:10.1371/journal.pone.0230224)
Supplement: Attachment — Submitted filename: response_to_reviewer_submission.docx [file pone.0230224.s002.docx]

We appreciate your advice. The manuscript has been revised as follows.

**Response to the editor**

**Comment 1:** Please ensure that your manuscript meets PLOS ONE's style requirements, including those for file naming. The PLOS ONE style templates can be found at <https://journals.plos.org/plosone/s/file?id=wjVg/PLOSOne_formatting_sample_main_body.pdf> and <https://journals.plos.org/plosone/s/file?id=ba62/PLOSOne_formatting_sample_title_authors_affiliations.pdf>

**Response 1:** We have ensured that the manuscript meets PLOS ONE's style requirements.

**Comment 2:** We note that Figures 1 and 2 include an image of a [patient / participant / in the study]. As per the PLOS ONE policy (http://journals.plos.org/plosone/s/submission-guidelines#loc-human-subjects-research) on papers that include identifying, or potentially identifying, information, the individual(s) or parent(s)/guardian(s) must be informed of the terms of the PLOS open-access (CC-BY) license and provide specific permission for publication of these details under the terms of this license. Please download the Consent Form for Publication in a PLOS Journal (http://journals.plos.org/plosone/s/file?id=8ce6/plos-consent-form-english.pdf). The signed consent form should not be submitted with the manuscript, but should be securely filed in the individual's case notes. Please amend the methods section and ethics statement of the manuscript to explicitly state that the patient/participant has provided consent for publication: “The individual in this manuscript has given written informed consent (as outlined in PLOS consent form) to publish these case details”.　If you are unable to obtain consent from the subject of the photograph, you will need to remove the figure and any other textual identifying information or case descriptions for this individual.

**Response 2:** We downloaded the Consent Form for Publication and the signed consent form is filed securely with our research documents. We amended the “Ethics Statement” in the “Methods” section to include the statement you provided.

Page 6, Lines 82-84

The individual in this manuscript in Figures 1 and 2 has given written informed consent (as outlined in PLOS consent form) to publish the case details.

**Comment 3:** Please include captions for your Supporting Information files at the end of your manuscript, and update any in-text citations to match accordingly. Please see our Supporting Information guidelines for more information: http://journals.plos.org/plosone/s/supporting-information.

**Response 3:** We included caption for the supporting information file at the end of the manuscript but before the references.

**Response to the reviewers**

**Reviewer #1:** The authors investigated the relationship between tongue thickness and tongue pressure in 254 Japanese elderly persons. The structure is adequate, the methods/results are well-written. Some points have to be addressed.
**Comment 1:** Reduce the size of the introduction. Focus on the importance of the topic and why it is important to conduct this study.

**Response 1:** We focused the introduction on the importance of the topic and deleted the sentences containing general information. Because another reviewer recommended adding an explanation of swallowing evaluation and tongue pressure to the introduction, we added the explanation.

**Comment 2:** Some points of the discussion (sentences about aspirations, etc.) need to be replace in the introduction to shed light the importance of the topic. Please, in the discussion only focus of the discussion of your results.

**Response 2:** We revised the introduction to shed light on the importance of the topic. We also rewrote the discussion section to focus solely on the discussion of our results.

**Comment 3:** The main weakness of this study is the lack of swallowing and aspiration evaluations. It's interesting to study the tongue size & pressure but it's needed for a practical idea. Here, there were no relationship with dysphagia or aspiration, which seem to be very important point associated with elderly frailty and morbi-mortality in case of disorder.

**Response 3:** As we mentioned in the limitations, we could not perform gold standard methods such as VF because the subjects were outwardly almost healthy patients. However, we evaluated participants using the ‘Food Intake LEVEL Scale (FILS),’ which is a scale for oral intake. We added the FILS as baseline data and re-analyzed the results. We show the relationship between FILS and baseline data in Table 3, and explained the relationship in the “Results” section. Below is the relevant text from the “Methods” and “Results” sections:

Page 10, Lines 159-162

Additionally, swallowing was evaluated using the Food Intake LEVEL Scale (FILS), which was administered by two physicians (YH and HM) [18]. FILS is a 10-point observer-rating scale to measure the severity of swallowing dysfunction, with lower scores indicating higher levels of dysfunction. Its convergent validity and intra-rater and inter-rater reliability have been established with the Functional Oral Intake Scale.

Page 15, Lines 237-240

Finally, the potential factors associated with the FILS scores were evaluated using multifactorial regression analysis by sex. In both the male and female group, only tongue pressure was significantly associated with the FILS scores (male; adjusted R^2^ = 0.592, *p* < 0.001, n = 163: Table 3A, female; adjusted R^2^ = 0.764, *p* < 0.001, n = 9: Table 3B).

**Reviewer #2:** This study is very interesting. The authors evaluate the thickness of the tongue as an expression of “oral frailty” and as possible swallowing disorder. I think that the role of tongue in dysphagia should be better described in the text. In particular describe how a reduced tongue strength can cause inhalation risk.
The statistical analysis been performed appropriately and rigorously. It would be interesting to know if the sample size estimation and power analysis was calculated. The limitations of the study are well described.

In this study, the authors evaluate the thickness of the tongue as an expression of “oral frailty” and as possible swallowing disorder.

**Abstract.**

**Comment 1:** Page 3, lines 33, 34. The sentence: “The tongue ultrasonography provides a less invasive technique for determining tongue thickness and related aspiration risks for elderly patients”. The authors should better explain or change the second part of this claim. Does the tongue ultrasonography provide a technique for determining aspiration risks?

**Response 1:** We appreciate your suggestion. We rewrote the sentence as follows.

Page 3, lines 38-40

We conclude that tongue ultrasonography provides a less invasive technique for determining tongue thickness and predicts oral frailty for elderly patients.

**Introduction.**

**Comment 2:** Page 4, lines 56, 57. VF is not performed in some centers, but currently it is performed routinely in many hospitals. You should change the sentence: “…However, VF is a specialized method that is not routinely performed with the general population...”.

**Response 2:** We appreciate your suggestion. We rewrote the sentence as follows.

Page 4, Lines 53-54

However, VF poses several problems because it requires equipment and carries the risk of radiation exposure and aspiration.

**Comment 3:** Page 4, lines 57, 58. “Hence, several simple and non-invasive techniques have been developed for evaluating swallowing function…”. A non invasive techniques for evaluating swallowing function is the Fiberoptic Endoscopic Evaluation of Swallowing (FEES) that is a gold standard for evaluation of pharyngeal phase of swallowing. You should add this exam with its bibliography to the introduction.

**Response 3:** We appreciate your suggestion. We rewrote the sentence as follows.

Pages 4-5, Lines 54-59

A fiberoptic endoscopic evaluation of swallowing is also a gold standard method, which is routinely performed by otolaryngologists or rehabilitation doctors. However, many general physicians do not acquire the skills required to perform the examination. Hence, several simple and non-invasive techniques have been developed for evaluating swallowing function, such as those for measuring tongue pressure and tongue thickness.

**Comment 4:** Page 4, lines 59, 60. You should describe some instrumental methods of measuring the tongue pressure with the relative bibliography.

**Response 4:** We appreciate your suggestion. We added the description as follows.

Pages 4-5, Lines 59-63

For measuring tongue pressure, the instruments are mainly divided into the sensor sheets type and the balloon type. The sensor sheets-type device is attached to the hard palate of patients and they actually swallow liquid or food [9]. For the balloon-type device, tongue pressure is measured by having a patient raise the tongue and push on the hard palate [6-8].

**Mathods. Subjects.**

**Comment 5:** Page 5, lines 82-85. What are the inclusion and exclusion criteria? What does it mean: “chronic disease”? “We excluded patients who had a history of otorhinolaryngologic disease”. Which ENT diseases have been excluded?

**Response 5:** Chronic diseases are lifestyle-related diseases (such as hypertension, diabetes mellitus, dyslipidemia). Otorhinolaryngologic disease is a pharyngeal or laryngeal tumor.

We rewrote the inclusionary and exclusionary criteria:

Page 6, Lines 87-91

We included those patients who consented to participate and were aged 65 years and older with lifestyle-related diseases (such as hypertension, diabetes mellitus, dyslipidemia). We excluded patients who had a history of otorhinolaryngologic disease (pharyngeal or laryngeal tumor) and/or neurodegenerative disease. Patients with paralysis were also excluded.

**Statistical analysis.**

**Comment 6:** In your study how the sample size estimation and power analysis was calculated?

**Response 6:** We appreciate your suggestion about statistical methods to make solid conclusions. We calculated the sample size according to the past investigations for the tongue thickness and tongue pressure in amyotrophic lateral sclerosis. We rewrote sentences in the “Statistical analysis” subsection of the “Methods” section as follows.

Pages 10-11, Lines 167-170

We calculated the required sample size according to past investigations for the tongue thickness and tongue pressure in amyotrophic lateral sclerosis [11,13]. Based on an alpha level = 0.05, and power = 0.80, we estimated that we would require a total of n = 212 participants.

**Discussion.**

**Comment 7:** Page 15, lines 250, 251. “One serious concern related to oral frailty is that elderly people often developed aspiration pneumonia”. You should better explain how oral frailty can develop aspiration pneumonia. What are the pathophysiological mechanisms? Can oral frailty cause aspiration pneumonia even in elderly people without neurodegenerative diseases? Is this pathological mechanism present in presbyphagia? The alteration of the oral phase of swallowing and the consequent premature spillage, can be the cause of increased risk of aspiration in the elderly people? You should better explain these mechanisms and add the related references.

**Response 7:** We appreciate your suggestion. We added an explanation of the relationship between oral frailty and aspiration pneumonia in the “Discussion” section.

Page 18, Lines 273-279

One serious concern related to oral frailty is that elderly people often developed aspiration pneumonia. Aspiration pneumonia is caused by many factors such as oral environment, swallowing dysfunction, decreased cough reflex, and immunodepression. These factors are associated with oral frailty [19]. Additionally, decreased tongue strength is associated with sarcopenia and contributes to oral frailty [20]. A reduction in tongue strength has been associated with an increased risk for aspiration as it increases the likelihood of bolus retention in the pharynx [21].
