# Supplementary material for: Tongue thickness measured by ultrasonography is associated with tongue pressure in the Japanese elderly
Source: PLoS One. 2020 Aug 7;15(8):e0230224. doi: 10.1371/journal.pone.0230224 (PMC7413413; doi:10.1371/journal.pone.0230224)
Supplement: Attachment — Submitted filename: R2_response_to_reviewer_submission.docx [file pone.0230224.s003.docx]

We sincerely appreciate the reviewer’s advice. Based on such suggestions, the manuscript has been revised as follows.

**Reviewer #3:**

This is a very interesting manuscript with a good scientific method to develop. Statistics are ok. All data are provided properly, and language is correct. All data underlying the findings described in their manuscript are fully available without restriction. Language is correct clear and unambiguous. I would like to introduce some issues to take into account with authors.

**Response to the reviewer**

**Comment 1:** line 91 introduction what kind of paralysis? Patients with tongue tie could not perform this test properly so they should be excluded. otorhinolaryngologic disease (pharyngeal or laryngeal
tumor) (what happens with maxylofacial tumor if there is any trismus present you can not do the test. It will be beter history of head and neck neoplams.

**Response 1:** We appreciate this comment. By ‘paralysis,’ we meant extremity paralysis, which is indicated in the manuscript (e.g. Line 92). Upon your suggestion, we excluded these patients from the study. Furthermore, we described the exclusion criteria in detail as follows:

Page 6, Lines 89-93: We excluded patients who had a history of otorhinolaryngologic disease (brain, facial, neck, pharyngeal, or laryngeal tumors) and/or neurodegenerative disease. Patients with oral abnormalities, such as tongue-tie; maxillofacial dysfunction, such as trismus; and those with extremity paralysis were also excluded.

**Comment 2:** Patients with sleep apnea syndrome had a different behavior with your conclusions. They have the lowest strength in the tongue and are correlated with increase tongue thickness. I will recommend you to read and include some reference to. Wang SH, Keenan BT, Wiemken A, et al. Effect of Weight Loss on Upper Airway Anatomy and the Apnea-Hypopnea Index. The Importance of Tongue Fat. Am J Respir Crit Care Med. 2020;201(6):718-727 doi:10.1164/rccm.201903-0692OCO' Connor-Reina C, Plaza G, Garcia-Iriarte MT, et al. Tongue peak pressure: a tool to aid in the identification of obstruction sites in patients with obstructive sleep apnea/hypopnea syndrome. Sleep Breath. 2020;24(1):281-286 doi:10.1007/s11325-019-01952-x. This kind of patients should be excluded from your study, or you should do some special emphasis in the discussion. OSAHS is another lifestyle related diseases commonly affect older patients. Your sample has normal BMI if they were overweight, do you think your conclusions will be fullfilled?.

**Response 2:** We thank the reviewer for these suggestions. There were 3 subjects in our study whose BMI was over 30 kg/m^2^ and who were not diagnosed with OSAHS. However, we did not perform a polysomnography on all subjects. Thus, this was indicated as a limitation of the study in the Discussion section. Upon your suggestion, we referred the articles recommended in our manuscript. We also considered racial difference, which was mentioned in the Discussion section.

Page 20, Lines 326-332: Third, we could not evaluate sleep apnea in the subjects of this study. It has been reported that people with sleep apnea have decreased tongue strength and increased tongue thickness due to excessive fat deposition, which causes tongue collapse [24,25]. In the present study, there were 3 subjects whose BMI was over 30 kg/m^2^ and were not diagnosed with obstructive sleep apnea hypopnea syndrome (OSAHS). However, we did not perform a polysomnography on all subjects. In future research, sleep apnea should be considered when studying the swallowing function.

**Comment 3:** There are many publications with IOPI to measure tongue strength in the world literature. I suggested you could include some of them. Their conclusions are similar as yours in the relationship with tongue strenght and age.

**Response 3:** We appreciate this suggestion. We mentioned the IOPI in the manuscript as follows:

Page 18, Lines 277-282: There are three main types of balloon-based tongue pressure measurement devices: the KayPENTAX device, the Iowa Oral Performance Instrument (IOPI), and the JMS Co. device. The JMS device, which is commonly used in Japan, shows lower values than the other balloon-based devices, but said values can be correlated linearly [6]. The IOPI is often used internationally, and shows the relationship between tongue pressure and age, which is consistent with our findings [19].
